# Naturalist's Monthly Report

**Published:** 1811-06

**Authors:** 


### NATURALIST'S MONTHLY REPORT.

### APRIL.

### BUDDING MONTH.

Emblem of life! see changeful April sail
In varying vest along the shadowy skies,
Now bidding summer's softest zephyrs rise,
Anon recalling winter's stormy gale,
And pouring from the cloud her sudden hail.

On the 1st of the month the wind was south-east; on the 2d westerly; on the 3d north-west, and afterwards south-west; on the 4th and 5th easterly; on the 6th east, and south-east; on the 7th north-east; on the 8th and 9th easterly; on the 10th variable; from the 11th to the 16th westerly; in the afternoon of the 17th south; on the 18th west; on the 19th south-east, and afterwards south-west; on the 20th and 21st southerly; on the 22d south-east; on the 23d and 24th south-west; on the 25th variable; on the 26th and 27th westerly; on the 28th south, and south-west; and on the 29th and 30th westerly.

There have been very few stormy days during the present month. On the 12th we had strong winds; in the morning of the 19th fresh gales, and in the following night a heavy squall with rain. The night of the 20th was also squally; and the morning of the 29th stormy.

We had rain, more or less, on the 4th, 6th, 7th, 10th, 13th, 16th, 18th, 19th, 20th, 26th, 28th, 29th, and 30th.

April 3d. This was a beautifully bright and warm day. The bloom buds upon the fruit trees have been considerably enlarged within the last few days, and promise a great profusion of blossom.

April 4th. Fine, gentle rain nearly the whole day.

April 6th. Some of the swallow tribe are arrived. Three or four were seen flying about near the surface of the river, apparently in pursuit of insects. The great body of swallows and martins will not, however, make their appearance probably in much less than a week from this time.

In the nights of the 7th, 8th, and 9th, we had very sharp frosts, which will tend greatly to check the progress of vegetation. It may be fortunate for the ensuing fruit season that very little indeed of the bloom is yet expanded.

April 11th. Gudgeons spawn.

April 13th. The cuckoo-flower *(cardamina pratensis)*, wall-flower *(cheiranthus cheiri)*, dog's mercury *(mercurialis perennis)*, wild strawberry *(fragaria vesca)*, cowslip *(primula veris)*, ground ivy *(glecoma hederacea)*, red nettle *(pedicularis sylvatica)*, and harebell *(Scilla nutans* of Smith) are now in flower.

April 17th. The cherry-tree is in bloom. Gooseberry and currant trees are in full leaf, as are likewise the elder and lime. The leaves of the hazel, and the sloe and hawthorn appear. The swallows and martins are all arrived.

April 18th. The mountain ash is in leaf.

April 19th. Apple-trees are in bloom. Young rooks are heard, and the titlark sings.

April 20th. The hedges are beginning to appear green. The flower-buds of the hawthorn are seen.

April 21st. There was much lightning in the night.

April 23d. This was a fine and hot day, in every respect like what we have in the middle of summer, the want of verdure and foliage excepted. Flesh-flies buzz about; and the common house-flies are nearly as numerous as in the summer.

The flowers of sheep's sorrel *(rumex acetosella)*, and ribwort plantain *(plantago lanceolata)*, colour all the dry sandy pastures.

Germander speedwell *(veronica chamædrys)*, procumbent speedwell (*agrestis*), sweet-scented vernal-grass (*anthoxanthum odoratum*), clammy mouse-ear (*cerastium viscosum*), upright pearlwort (*sagina erecta*), and soft-leaved crane'sbill (*geranium molle*) are in flower.

*Elater cinereus*, and several species of moths of the subdivision *tinea*, appear. Female wasps, also, now fly about.

April 25th. Hedge roses are in leaf. The May-fly, and some species of *phryganea* have issued from their chrysalids.

Perch have retired to the smooth waters to spawn among the weeds.

April 28th. So powerful were the sun-beams in the middle of this day, that sheep were compelled to retire into the shade.

April 30th. Cock-chaffers fly in the evening.

There has been much rain in the country westward of us. The rivers are muddy, and in some places out of their banks.

*Hampshire.*

